# The Phospholipid Research Center: Current Research in Phospholipids and Their Use in Drug Delivery

**DOI:** 10.3390/pharmaceutics12121235

**Published:** 2020-12-18

**Authors:** Simon Drescher, Peter van Hoogevest

**Affiliations:** Phospholipid Research Center, Im Neuenheimer Feld 515, 69120 Heidelberg, Germany; pvh@phospholipid-research-center.com

**Keywords:** phospholipids, lecithin, drug delivery, parenteral, oral, topical

## Abstract

This review summarizes the research on phospholipids and their use for drug delivery related to the Phospholipid Research Center Heidelberg (PRC). The focus is on projects that have been approved by the PRC since 2017 and are currently still ongoing or have recently been completed. The different projects cover all facets of phospholipid research, from basic to applied research, including the use of phospholipids in different administration forms such as liposomes, mixed micelles, emulsions, and extrudates, up to industrial application-oriented research. These projects also include all routes of administration, namely parenteral, oral, and topical. With this review we would like to highlight possible future research directions, including a short introduction into the world of phospholipids.

## 1. Introduction

**Figure 1:**
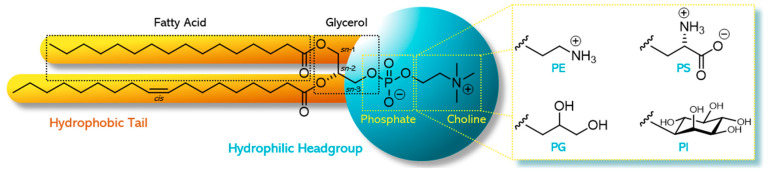
Chemical structure of a phospholipid as exampled by 1-palmitoyl-2-oleoyl-*sn*-glycero-3-phosphocholine (POPC) and the different alternative headgroups varying in the type of alcohol: phosphatidylethanolamine (PE) with ethanolamine, phosphatidylglycerol (PG) with glycerol, phosphatidylserine (PS) with serine, and phosphatidylinositol (PI) with inositol.

Phospholipids are unique and versatile molecules. They are of natural occurrence and the main components in cellular membranes. Arranged as a lipid bilayer, phospholipids play a significant role in the structure and functionality of biological membranes. They are amphiphilic and consist of a hydrophilic headgroup and a lipophilic/hydrophobic tail (Figure 1).

In phospholipids, the *sn*-1 and *sn*-2 position of the glycerol backbone are esterified with fatty acids of varying length and degree of saturation. The remaining *sn*-3 position is esterified with phosphoric acid, which, in turn, is esterified with an alcohol [1]. Depending on the structure of this alcohol, different types of phospholipids comprise, for example, phosphatidylcholine (PC), phosphatidylethanolamine (PE), phosphatidylglycerol (PG), phosphatidylinositol (PI), or phosphatidylserine (PS) [2]. The specific and non-random distribution of substituents over the positions *sn*-1, *sn*-2, and *sn*-3 of the glycerol introduces chirality. Depending on the structure of the polar headgroup and pH of the surrounding medium, PE and PC are zwitterionic and have a neutral charge at pH 7, whereas PG, PI, and PS are negatively charged at this pH value. As an example, the chemical structure of 1-palmitoyl-2-oleoyl-*sn*-glycero-3-phosphocholine (POPC) and the different types with alternative headgroups are shown in Figure 1.

The physiological functions of phospholipids are manifold. For instance, besides their functional role in cell membranes, phospholipids, mainly PC, have digestion/metabolic functions in bile (as monoacyl-phospholipids, i.e., lyso-phospholipids) to solubilize cholesterol and fatty components in food and lipophilic drug substances [3]. Moreover, phospholipids act as lipoprotein components for transport of fat between gut and liver, as source for acetylcholine (in the case of PC), and as source of (essential) fatty acids and energy [4]. In addition, in lung surfactant, a specific phospholipid, namely 1,2-dipalmitoyl-*sn*-glycero-3-phosphocholine (DPPC), occurs [4], lowering the surface tension at the air/water interface within the alveoli of the lung. PS is a component of the lipid–calcium–phosphate complex for deposition during bone formation [5], and it is active in regulation of blood coagulation [6] and apoptosis [7].

Due to their physiological roles, phospholipids possess a very low toxicity profile and can be used for any route of administration [8]. The structural diversity of phospholipids and the resulting variability of chemical, biophysical, and technological properties leads to wide use of phospholipids in various pharmaceutical formulations [9,10,11,12,13]. They can be technologically used as an emulsifier, wetting agent, solubilizer, and agent in the formation of liposomes and mixed micelles, to name but a few. From a pharmaceutical perspective, they are used as key excipients for parenteral administration for solubilizing formulations such as liposomes, mixed micelles, and oil-in-water (o/w) emulsions; in liposomes for drug targeting and slow release; and for topical administration to the lung and the skin for slow release and enhanced skin interaction, respectively. After oral administration, phospholipids are used to suppress gastrointestinal (GI) side effects of, for example, non-steroidal anti-inflammatory drugs (NSAIDs) and explored as solubilizers to enhance the oral absorption of poorly water-soluble compounds. Nevertheless, phospholipids are still not widely applied as excipients for the development and manufacture of pharmaceuticals, although they represent, as natural compounds, for instance, very effective alternatives to synthetic (non-natural) emulsifiers such as polysorbates, polyoxyethylene castor oil derivatives, and sucrose esters. To change this, renowned international scientists conducting research on phospholipids founded the Phospholipid Research Center Heidelberg (PRC) in 2006 with the support of Lipoid GmbH (Ludwigshafen, Germany) and PHOSPHOLIPID GmbH (Cologne, Germany) [14]. The main vision of the PRC is to translate the physiological and physicochemical properties and benefits of phospholipids into their optimal pharmaceutical use as excipients.

**Figure 2:**
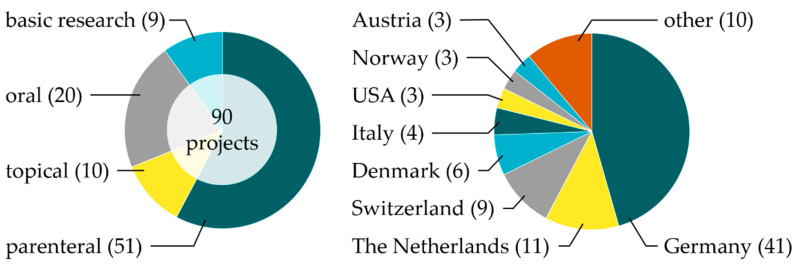
Distribution of the 90 research projects supported so far by the Phospholipids Research Center Heidelberg (PRC) regarding the phospholipids’ administration type/basic research (**left**) and location of projects across the world (**right**), with the number of projects in parentheses.

Since the foundation of the PRC, 90 research projects related to phospholipids have been supported as of 2020. In this comprehensive review, we classify the projects according to the main application route of phospholipid-based formulations in parenteral (51), oral (20), and topical projects (10). In addition, nine projects related to basic phospholipid research are included. The distribution of the projects according to their main application route/basic research as well as the location of the institutes of the principal investigator is provided in Figure 2. Besides the countries mentioned, the term “other” stands for one project each from Croatia, Finland, France, Iran, Israel, Nigeria, Poland, Portugal, Sweden, and the United Kingdom, i.e., 46% of all projects are in Germany, 48% in Europe (without Germany), and 6% in the rest of the world. This review gives an overview of current (drug delivery-related) phospholipid research and highlights possible future research tendencies [15].

However, before performing research with phospholipids, their quality (sources, purification, and resulting purity) and the intended use should be taken into consideration. Depending on the type of application of the phospholipids, there are different requirements to prove their pharmaceutical quality.

## 2. Phospholipids—General Aspects

First, one must be aware of the different meanings and uses of the term “phosphatidylcholine” and “lecithin”. According to the United States Pharmacopeia (USP), lecithin is described as “a complex mixture of acetone-insoluble phosphatides (i.e., phospholipids), which consist chiefly of PC, PE, PI, and phosphatic acid (PA), present in conjunction with various amounts of other substances such as triglycerides, fatty acids, and carbohydrates, as separated from the crude vegetable oil source. It contains not less than 50% of acetone-insoluble matter.” Unfortunately, especially in the American literature, lecithin is also used as a synonym of PC, which is the pure compound. Descriptions of the phospholipid component in products as “lecithin” leaves open which lipid is used. We therefore recommend using for natural phospholipids the term “lecithin” only when the product contains less than 80% by weight total phospholipids and the term “phospholipid” when the product contains 80–100% by weight phospholipids (e.g., PC, PE); the specific phospholipid should be mentioned when the product contains more than 90% by weight of that specific phospholipid.

### 2.1. Natural Phospholipids

In principal, one can distinguish between natural and synthetic phospholipids. Natural phospholipids can be obtained from vegetable sources such as soybeans, sunflower, rape (canola) seed, wheat germ, and flax seed; and animal material such as hen egg yolk, milk, or krill. The phospholipid and fatty acid profiles of the various lecithins depend, of course, on the raw material sources. The composition of de-oiled vegetable lecithin from variable sources and egg lecithin, respectively, are provided as example in Table 1. Besides PC, PE, and PI, other components can also occur in natural lecithins, namely sphingomyelin (SM), PA, and minor amounts of phospholipids containing only one acyl chain in the *sn*-1 position, namely lyso-phosphatidylcholine (LPC) and lyso-phosphatidylethanolamine (LPE). In all cases, PC is the main phospholipid component. To convert these materials into high-quality excipients, meeting pharmacopeial and regulatory requirements for parenteral and other specific formulations, extraction and chromatography procedures are applied (see Section 2.3). 

The fatty acid composition of typical batches of vegetable de-oiled lecithins and egg phospholipid of different PC content, respectively, is given in Table 2 [9]. The corresponding fatty acids are listed in their short notation, with the first digit indicating the number of carbon atoms and the second digit the numbers of *cis*-double bonds: C14:0, myristic acid (tetradecanoic acid); C16:0, palmitic acid (hexadecenoic acid); C18:0, stearic acid (octadecanoic acid); C18:1, oleic acid (octadecenoic acid); C18:2, linoleic acid (octadecadienoic acid); C18:3, α-linolenic acid (ALA, octadecatrienoic acid); C20:0, arachidic acid (eicosanoic acid); C20:4, arachidonic acid (ARA, eicosatetraenoic acid); C22:0 behenic acid (docosanoic acid); C22:4, docosatetraenoic acid; C22:5, docosapentaenoic acid (DPA); and C22:6, cervonic acid (docosahexaenoic acid, DHA). The presence of the polyunsaturated fatty acids (PUFAs) C20:4 and C22:6 is typical for hen egg yolk phospholipids.

### 2.2. Synthetic Phospholipids

Due to the presence of unsaturated fatty acids in natural phospholipids, the liquid crystalline to gel phase transition temperature (*T*_m_) is below 0 °C. These phospholipids are at ambient temperature in the liquid crystalline state (*L*α phase) and form upon hydration flexible structures/mesophases [2], suitable for specific pharmaceutical technological applications. In some formulations, however, when, for example, more physically stable liposomes with increased stability in blood plasma or phospholipids with more powder-like properties are required, phospholipids with higher *T*_m_ are preferred [18]. Phospholipids with saturated fatty acids possess these properties and can be obtained by hydrogenation of natural phospholipids with unsaturated fatty acids [19], resulting in, for example, HSPC (hydrogenated soybean PC). The progress of the hydrogenation can be followed by monitoring the iodine value, which is a measure of the degree of unsaturation of fatty substances.

Nowadays, alternative biochemical synthesis routes via enzyme-catalyzed reactions serve as a viable alternative to organic-chemical synthesis steps. In that respect, the use of enzymes for phospholipid modification has moved quickly in recent years, not only in academic research but also in industry [20]. The fast-growing use of enzymes for polar lipid modification arises from factors such as milder reaction conditions, less environmental pollution, better specificity for improved quality, and higher efficiency of reactions. Specific enzymes are suitable for different modification purposes to modify/synthesize phospholipids. For acyl modifications, natural enzymes such as phospholipase A1 and A2 (PLA1 and PLA2), which selectively cleave the fatty acid in *sn*-1 and *sn*-2, respectively, leading to lyso-phospholipids, can be used. Phospholipase B (PLB) is an enzyme with a combination of both PLA1 and PLA2 activities; that is, it can cleave acyl chains from both the *sn*-1 and *sn*-2 positions. Phospholipase C (PLC) can hydrolyze the bond between the glycerol oxygen and the phosphate group, leading to the formation of diacylglycerol and phosphocholine. Unfortunately, this enzyme family is only active for the hydrolysis reaction and not for the reformation process. Therefore, PLC and PLB are not used industrially. Finally, phospholipase D (PLD) is the only potential enzyme for polar group modification. This enzyme cleaves the bond between the phosphate and the choline. Examples of enzyme-modified (“semi-synthetic”) natural phospholipids prepared from natural PC are lyso-PC, soybean PE, soybean PG, egg PG, and their saturated analogs.

To study more mechanistic biochemical or biophysical aspects of phospholipids at the molecular level in natural environments or in model membranes, various synthetic approaches to chemically well-defined phospholipids have been developed. These “full-synthetic” phospholipids are then homogeneous with respect to the polar headgroup and fatty acid composition [2,20,21]. This aspect has been reviewed by Hoogevest and Wendel [9], and we refer to this review and the literature cited therein. In addition to the need for synthetic analogs of natural phospholipids, further synthetic phospholipids were designed to, for example, optimize the drug targeting properties of liposomes. Examples are the PEGylated phospholipids, which are phospholipids bearing a polyethylene glycol (PEG) chain of variable length attached to their headgroup [22], and the cationic phospholipid 1,2-diacyl-P-*O*-ethylphosphatidylcholine [23], to name but a few.

For the preparation of “full-synthetic” phospholipids, several starting compounds are conceivable. The classical approach to synthesize diacyl-PCs is to start from d-mannitol. However, this synthetic method is lengthy, requires toxic chemicals and solvents in large excess, and reaction intermediates may be unstable and subject to partial racemization [24,25]. A simpler method to synthesize symmetrical glycerophospholipids, which are phospholipids bearing two identical fatty acids, is a method starting from (*R*) or (*S*) glycidyl tosylates [26]. The enantiomerically pure glycidyl derivative is, however, expensive, making large scale syntheses of glycerophospholipids impractical [2]. Another route to produce phospholipids with defined fatty acids is to start from glycerophosphocholine (GPC), which is also termed *sn*-glycero-3-phosphocholine, l-α-glyceryl-phosphorylcholine, α-GPC, or choline alfoscerate. GPC can be synthesized in an enantioselective manner using a biotransformation procedure based on the phosphorylation of glycerol by adenosine triphosphate (ATP) catalyzed glycerol kinase [27] or, preferably, produced by means of alkaline hydrolysis from natural PC maintaining the natural stereoisomeric structure. Starting from the latter, symmetrical phospholipids can be synthesized in a one step process using activated acyl derivatives, such as acylimidazolides or anhydrides [21,28], and various coupling reagents such as dicyclohexylcarbodiimide (DCC) in combination with 4-(dimethylamino)pyridine (DMAP) [29] or 2-methyl-6-nitrobenzoic anhydride (MNBA) [30]. For the further synthesis from GPC to asymmetrical, i.e., mixed fatty acid chain phospholipids, organic chemical methods as well as enzymatic procedures can be applied. Which synthesis route will finally be selected is, from an industrial perspective, dependent on the (commercial) availability of key intermediates or starting materials.

### 2.3. Industrial Production of Phospholipids

**Figure 3:**
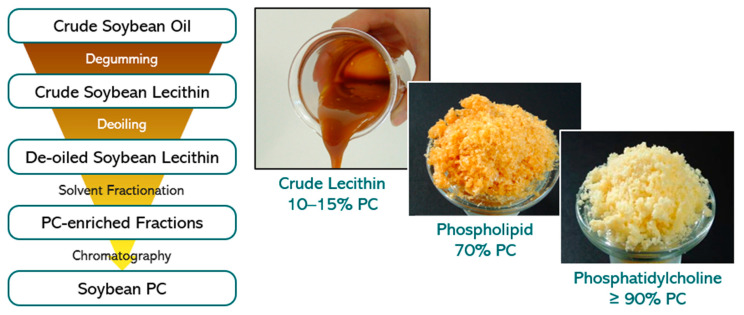
(**Left**) Flow chart of isolation process steps of soybean PC derived from crude soybean oil. (**Right**) Visual appearance of soybean lecithin/PC fractions with variable PC content.

To exemplify the production process of natural phospholipid excipients starting from plant oil, the production process of soybean lecithin is given. First, crude soybean lecithin is isolated by degumming of the crude soybean oil as obtained by extraction from soybeans [31] (Figure 3).

The crude soybean lecithin obtained serves as starting material for production at a large scale of soybean lecithin fractions with higher PC content. These fractions are obtained in high yields by extraction methods using the non-toxic solvents acetone and ethanol followed by chromatographic purification procedures and appropriate solvent removal methods. All solvents can be recycled and reused. By selecting appropriate sequential extraction and chromatography methods, several lecithin fractions differing in PC content from 20 to 80% up to pure PC (≥98%) and in ratios of phospholipids to non-polar lipids can be reproducibly achieved. This procedure applies to soybean oil and all vegetable oils used to produce lecithin, and high-purity phospholipids/PC. Furthermore, egg phospholipids are isolated from hen egg yolk with similar extraction and chromatography methods as for soybean lecithin.

### 2.4. Regulatory and Safety Aspects

Natural phospholipids are in general well known to regulatory authorities. Moreover, their track record as excipients with very high tolerability and biocompatibility is outstanding. The World Health Organization (WHO) places no limit on the oral intake of lecithin. Furthermore, no limit for the value of acceptable daily intake (ADI) for lecithin as a food additive is given [32]. The pediatric oral use of phospholipids (soybean) is in general allowed, of course with precautions for soybean allergy (see below). Alternatively, sunflower phospholipids, with no allergy warnings, can be used. After parenteral administration, egg and soybean lecithin—unsaturated and saturated variations—are well tolerated.

The European Commission declares that lecithin is a food additive (E322) “generally permitted for use in foodstuffs” [33]. Furthermore, no ADI value has been fixed for lecithin in Europe; the material may be used quantum satis [34]. The US Food and Drug Administration (FDA) assigned the generally recognized as safe (GRAS) affirmation for lecithin [35] and enzyme-modified lecithin [36].

Although phospholipids on their own do not have an allergy potential, phospholipids derived from soybean and hen egg yolk must be labelled as potentially allergenic because of the soy and egg origin [37,38]. It is known that these allergies are caused by residues of soy and egg proteins. In purified soybean phospholipids used for pharmaceutical application, the protein residues were found to be below the lower limit of detection (LLOD) of a soybean specific enzyme-linked immunosorbent assay (ELISA), which was 1 ppm [39]. The egg protein content of purified egg lecithin for parenteral administration was tested by Lipoid GmbH [40]. The most sensitive immunological detection method of proteins showed less than the LLOD of 0.5 ppm of egg protein in purified egg phospholipids. Since, however, from an immunological perspective one molecule of soy protein or egg protein represents a theoretical risk, still the origin of such products has, according to regulatory authorities, to be labelled to alert allergic individuals. In a recent study on a propofol emulsion with soybean oil and egg phospholipids as emulsifier and its parenteral use in children with allergies to egg, peanut, soybean, or other legumes, it was concluded that genuine serious allergic reaction to product was rare and is not reliably predicted by a history of food allergy [41].

### 2.5. Use of Phospholipids in Pharmaceutical Formulations

Phospholipids—natural as well as synthetic—are broadly used in pharmaceutical technology as wetting agents, emulsifiers, and builders or components of different lipid mesophases such as liposomes, micelles, mixed micelles, inverted micelles, cubosomes, etc. [9]. These functional properties are applied in many types of pharmaceutical formulations such as suspensions, different types of emulsions, solid dispersions, lipid nanoparticles, drug/phospholipid complexes, etc. [42,43]. With respect to their physiological role, phospholipids possess a very low toxicity profile and can be used for any route of administration, namely parenteral, oral, and topical. Regarding the versatility, phospholipids are superior excipients compared to synthetic non-biodegradable polymers, which are not suitable to be used for every administration route and which are, by definition, un-physiological. In case natural phospholipids derived from hen egg yolk or soybean are selected, attention should be paid to the minimal quality in phospholipid content, which depends on the administration route. For oral and dermal administration, natural phospholipids with at least 45% PC can be used, whereas for parenteral use, at least 70% PC is common [44]. For specific high-tech parenteral products (for injection), more expensive, synthetic, and chemically well-defined phospholipids of high purity may be the best choice, whereas for topical and oral administration (and of course other parental applications), cost effective natural phospholipids are better.

The categories of pharmaceutical formulations, which are of interest to study the occurrence of natural and synthetic phospholipids, respectively, are products used for parenteral administration [9]. These are mainly liposomes, o/w emulsions, mixed micelles for intravenous (i.v.) use and slow release and vaccine vehicles, and drug suspensions for intramuscular (i.m.) and subcutaneous (s.c.) administration. Liposomal products for i.v. administration are formulated with synthetic as well as natural phospholipids. Liposomes can also be used as vehicles for slow release after local parenteral administration, i.e., at the surgical site, epidural or intrathecal. In this case, mainly synthetic phospholipids are applied. Egg phospholipids are used in o/w emulsions for parenteral nutrition (for example Intralipid) [45] and as carriers for oil-soluble drug substances [46]. In mixed micellar formulations, including phospholipids and different choate salts, exclusively soybean phospholipids are used. Here, the phospholipids act as solubilizer for poorly water-soluble substances or as active principles (soybean PC), through the presence of PUFAs for treatment of liver disorders [47]. Considering finally products comprising phospholipids for pulmonary administration, natural and synthetic phospholipids are applied. Most of these products are used for respiratory distress syndrome in infants [48] or bacterial lung infections [49].

More details regarding the use of phospholipids in pharmaceutical formulations, including a variety of examples and drug products, can be found in the literature [8,9].

## 3. Phospholipids in Research—Project Overview

In the following sections, we give an overview about the research projects that have been approved since 2017 and are currently still ongoing or have recently been completed. There are 37 research projects, which can be subdivided into parenteral (23 projects), oral (7), and topical (4) administration/use of phospholipid-based formulations, and projects covering basic research (3).

### 3.1. Parenteral Administration

The most prominent mode of application of lipid-based formulations is the parenteral route, including i.v., i.m., and s.c. administration. Within this parenteral subgroup, liposomes and liposomal formulations, respectively, of active pharmaceutical ingredients (API) play an important role, which is due to but not restricted to the introduction of the Doxil^®^—the first nano-drug approved by the FDA in 1995 [50]. Although this is the largest set of projects, we present only a brief outline (Table 3). For projects approved prior to 2017, please refer to our homepage (www.phospholipid-research-center.com).

Over the last decades, liposomes have played—and they still do—a leading role in the field of nanomedicine [51]. However, there are several obstacles and barriers that need to be overcome before an API can reach its target and develop its pharmacological activity, for example the fast opsonization by the mononuclear phagocytic system (MPS) after i.v. administration [52,53]. Crommelin et al. describe in a very inspiring way which approaches should be focused on to promote the success of liposome-based formulations as a drug carrier and delivery system [51].

#### 3.1.1. Stimuli-Responsive Liposomes

One possibility to increase the effectiveness of liposomal drug delivery systems is the use of an external trigger to release the liposomal content at the targeted area. As external triggers, one may use focused ultrasound, light, or X-ray.

The group of Robbert Jan Kok (University Utrecht, Utrecht, the Netherlands) employed two types of stimuli-responsive nanocarriers to bring a plant-derived anti-cancer agent, mistletoe-lectin-I (ML-I) in the respective case, to the target cells. The entrapment of ML-I in liposomes protects the bioactive protein against degradation, while also preventing off-target effects caused by the toxin. Since ML-I cannot pass over the intact lipid membrane, two external stimuli were tested. Heat [54] induced a phase transition in the lysolipid-containing lipid membrane leading to bilayer destabilization and pore formation, and the ML-I could leave the cargo [55]. Secondly, ultrasound [56] also induced a disruption (cavitation) of the lipid bilayer, releasing the contents [57]. Since the formulation in ultrasound-sensitive liposomes (USL) showed the triggered release of over 90% compared to 10% release from temperature-sensitive liposomes (TSL), USL are therefore a promising approach, despite the complex purification procedure and the initial losses of the encapsulated cargo.

Another trigger can be light. Tatu Lajunen (University of Helsinki, Helsinki, Finland) combined a light activation technology based on indocyanine green [58,59] with a hyaluronic acid (HA) coating of liposomes. HA is an endogenous vitreal polysaccharide that potentially enables targeting to cluster of differentiation 44 (CD44), which is overexpressed on the surface of several cancer cells [60]. Moreover, the coating with HA increased the stability of liposomes in plasma when compared to PEGylated liposomes. With this combination, Lajunen was able to show a light-activated—using near-infrared (NIR) light—drug release in buffer, plasma, and vitreous samples [61]. Hence, these HA-coated light-activated liposomes seem to be a functional and promising alternative for intravenous and ocular drug delivery.

To improve the management of treatment-resistant cancers, there is a continued need for new strategies to augment radiotherapy outcomes. The use of ultra-small gold particles (nanoclusters) is a promising way to improve radiation absorption by cancer tissues [62,63], yet their therapeutic potential is limited because of poor tumor accumulation and permeation [64,65,66]. Here, the group of Mans Broekgaarden (University Grenoble, Grenoble, France) aims to engineer liposomes to overcome these limitations, thereby enabling the gold nanoclusters to hyper-sensitize cancer tissues to radiotherapy [66]. The liposomes contain oxidation-susceptible phospholipids, gold nanoclusters in their aqueous core, and novel phospholipid-anchored photosensitizers in their lipid bilayer. During photodynamic therapy or radiotherapy, these compounds generate high levels of reactive oxygen species that oxidize the liposomal lipids and release the gold nanoclusters in the tumor microenvironment [64,67]. Combining phospholipid-anchored photosensitizers with high-Z element nanoclusters to achieve radiotherapy-controlled drug release from liposomes is a ground-breaking novel concept that hopefully will be developed during this project. The oxidation-responsive liposomes (OXIL) thus carry the potential to augment various clinically relevant therapies that can benefit the treatment of a broad spectrum of cancer types.

Hejian Xiong from the University of Texas at Dallas (USA) is following a similar approach. He is using a highly photosensitive nanovesicle (liposome) to achieve repeatable and adjustable on-demand local anesthesia in superficial or deep tissues, which consists of gold nanoparticles attached to mechano-responsive liposomes. Mechano-responsiveness means that liposomes release their cargo upon mechanical stress such as shear stress [68]. A class of artificial phospholipids, namely 1,3-diamidophospholipids [69] synthesized by Andreas Zumbühl (now at Acthera Therapeutics AG, Basel, Switzerland) and his group, shows this remarkable property [70]. NIR laser pulses can activate the gold-coating to create nanomechanical stress to break the packing defects, leading to efficient cargo release [71,72]. The outcome of this study will not only be the development of a new kind of photosensitive phospholipid liposome but will also contribute to on-demand, personalized local anesthesia and pain management, since locally injected anesthetics present fast systemic absorption, leading to short duration and risk of systemic toxicity [73,74].

#### 3.1.2. Targeted Liposomes

The use of ligands, which are presented on the surface of liposomes to bring APIs specifically to the sites of action, is a longstanding endeavor for liposome researchers. Although positive results in animal models have been reported, no positive effects on the efficiency in patients have been published for ligand-targeted liposomes so far [51,75]. Recently, Belfiore et al. [76] and Wang et al. [75] reviewed the obstacles these ligand-based liposomes encounter upon injection.

Until encapsulated APIs are released from the liposomal container, the formulation has the pharmacokinetic properties of the nanoparticle and is therefore subject to the enhanced permeability and retention (EPR) effect. Due to the fenestration of endothelial cells in capillary vessels of malignant tumors, liposomes up to a size of 500 nm can diffuse into the tissue and accumulate there [77,78]. This passive, targeted drug release is the principle of most i.v.-administered liposomal formulations [79]. The size of the liposomes is not only decisive for the efficiency of the EPR effect, but also for the interaction of the liposomes with the body’s immune system. The larger the liposomes, the faster the phagocytic cells of the MPS are activated and the more interactions with plasma proteins take place [52,53].

The project of Luisa Corvo (University Lisbon, Lisbon, Portugal) focuses on liver reperfusion injury, which is a severe pathology with a very high unmet therapeutic need, as patients in whom a liver transplantation fails do not have any treatment option left [80]. The objective of the project is to improve the outcome of liver transplantation by using i.v. liposome therapy after transplantation to target two important pathologic pathways in liver perfusion ischemic injury: oxidative stress and inflammation. Liposomes offer the unique opportunity to load anti-inflammatory drugs in the interior while using the phospholipid bilayer for the encapsulation of anti-oxidant lipophilic compounds, including phospholipids composed of PUFAs [81,82].

The tumor microenvironment strongly contributes to aggravating tumor growth and causing treatment failure. Tumor-associated macrophages (TAMs), M2-type macrophages, are key cells in the tumor microenvironment, inducing tumor growth, invasion, and metastasis [83,84]. In this respect, the group of Jai Prakash (University Twente, Enschede, the Netherlands) developed TAM-targeting liposomes by introducing carboxylated phospholipids that bind to scavenger receptors expressed by TAMs. These TAM-targeted liposomes are applied to specifically target M2-modulating drugs, namely STAT6 inhibitor AS1517499 [85], zoledronic acid, and muramyl tripeptide (MTP-PE). Targeting of these inhibitors specifically modulates TAMs either by inhibiting M2-type macrophages or reversing them into tumor-suppressing M1-type macrophages—unraveling the biological significance in the tumor microenvironment. A follow-up study of this project (“Immunostimulating liposomes targeting M2 macrophage to eradicate cancer”—2020, ongoing) has been recently approved for funding.

In nature, biological membranes consist of more than fifty percent of (phospho)lipids. These lipids play a central role in cell–cell interactions and tissue recognition. Despite their important biological role, drug delivery systems rarely take advantage of the lipid profile to improve targeting. In fact, in most cases, proteins or sugar groups conjugated to the surface of lipid-based drug delivery systems are used to modulate cell uptake and biodistribution. In preliminary studies, the group of Avi Schroeder (Israel Institute of Technology, Haifa, Israel) found that the lipid profile of liposomes is enormously important for determining the biological fate of liposomes in cells of breast cancer. Schroeder will now study how lipids can be used to control tissue distribution [86,87,88], specifically to sites of breast cancer metastases in mice. A combinatorial barcoding apparatus will be used to study the biological fate of an array of liposomes of variable composition to triple negative breast cancer metastasis.

Pediatric sarcomas account for about 15% of pediatric cancers. These sarcomas often display a highly aggressive behavior with early tendencies for the development of metastasis. Although current treatment regimens, including surgery and chemotherapy, can achieve good response rates, the relapse rate is generally high with an extremely poor prognosis. The aggressive chemotherapies needed to fight relapsed tumors have significant toxicity, generating late side effects, a major complication in pediatric oncology [89]. Here, liposomal formulations can decrease systemic side effects by passive accumulation through the EPR effect, and the group of Michele Bernasconi (Bern University Hospital, Bern, Switzerland) aims to investigate the possibility to further increase local drug concentration by targeting liposomes to the tumor site. Selected ligands, for example peptides or nanobodies, showing high affinity for diseased cells or tissue, can be conjugated to drugs, macromolecules, or colloidal particles and bring their cargo to the tumor in a relatively selective fashion [90,91,92,93]. In the past years, Bernasconi et al. have identified peptides and nanobodies with strong affinities for rhabdomyosarcoma—the most common soft tissue sarcoma in children [94]. Moreover, they have optimized the formulation of peptide- and nanobody-targeted liposomes loaded with vincristine and will now identify the optimal liposomal formulation for targeted drug delivery to rhabdomyosarcoma. More efficient and less toxic therapies for pediatric sarcomas will hopefully increase survival rates and have a profound positive impact on the quality of life of pediatric sarcoma survivors.

Cancer accounts for 26% of all deaths worldwide. While the introduction of checkpoint inhibitors has dramatically changed the outlook of some cancers, only a fraction of all cancer patients benefits from these therapies, mainly due to a failed immune cell activation directed to the tumor [95]. Classical vaccination approaches have failed so far to boost sufficiently strong immune responses to combat cancer, and there is, hence, an urgent need for new strategies to be developed to induce effective anti-tumor immune responses [96]. In this respect, the group of Joke den Haan and Yvette van Kooyk (both University of Amsterdam, Amsterdam, the Netherlands) proposes the development of virus-like liposomes as a novel dendritic cell-targeted vaccination strategy in which they will use ligands of CD169 molecules expressed on antigen presenting cells, immune-activating viral-mimic molecules as adjuvant, and encapsulated tumor antigens [97,98]. Using a combination of in vitro assays and in vivo models, this study will investigate the efficacy of virus-like liposomes as novel nano-vaccine carriers that target CD169 antigen presenting cells for cross-presentation and tumor-specific T cell activation—to prolong survival and to contribute to a better quality of life of cancer patients.

The last project in this subgroup is related to drug delivery into the brain. Here, the blood–brain barrier (BBB) is a very selective biological barrier protecting the brain. It is formed by endothelial cells of microvessels being characterized by extremely tight junctions and high expression of export proteins. It is impermeable to macromolecules, including biologicals. However, biologicals are of highest interest for the therapy of Alzheimer’s disease [99]. A promising option to overcome the BBB with macromolecules is the use of colloidal drug carrier systems—such as, for example, liposomes [100]—or solid–lipid nanoparticles (SLNs), which are surface-modified to recognize specific binding sites at the BBB. Surface modifications could be achieved by coupling such nanocarriers to antibodies against receptors at the BBB, to cell penetrating peptides (CPPs), or to (fragments of) apolipoproteins. Only very few data are available with macromolecules, despite a very urgent clinical need for Alzheimer’s disease, as all clinical trials with conventional drugs have failed so far. In this context, Ulrike Müller (University Heidelberg, Heidelberg, Germany) aims to use liposome-mediated drug delivery for the transfer of macromolecules across the BBB. The Müller group expects to conduct proof-of-principle experiments to deliver biologicals across the BBB.

#### 3.1.3. Exosomes

Extracellular vesicles (EVs) are membrane-contained vesicles released in an evolutionally conserved manner by cells ranging from prokaryotes to higher eukaryotes and plants [101]. The importance of EVs lies in their ability to transfer information to other cells and thereby influence the function of the receiving cell [102]. EV-mediated signals can be transmitted by all the different biomolecule categories, including proteins, lipids, nucleic acids, and sugars, and the unique package of this information provides both protection and the ability to deliver several different messengers simultaneously, even to locations remote from the vesicular origin. EVs can be broadly classified into three main classes, with exosomes being one of them. Exosomes are formed within the endosomal network and are further released upon outward budding and fission of the plasma membrane [101,103].

Liver fibrosis is a reversible wound-healing response to repeated and chronic organ injury, characterized by the aberrant accumulation of the extracellular matrix. Scar formation may finally lead to organ dysfunction, cirrhosis, and hepatocarcinoma. The main profibrogenic cells of injured liver, namely hepatic stellate cells (HSC), following activation by specific stimuli undergo phenotypical changes towards myofibroblasts and contribute to the deposition of collagen [104]. To date, no specific anti-fibrotic drug treatment is available, although the positive action of certain hepatoprotectors has been acknowledged. Essential phospholipids have been used for decades as a supportive therapy for liver diseases, but a complete picture of their mechanism of action still needs to be drawn. Among the different molecular mechanisms involved in the progression of the disease, it has been suggested that EVs may play a pivotal intercellular role [105]. In this respect, the project of Paola Luciani (formerly University Jena, Jena, Germany, now University Bern, Bern, Switzerland) and Gregor Fuhrmann (Helmholtz Institute for Pharmaceutical Research Saarland, HIPS, Saarbrücken, Germany) aims at profiling EVs from a human immortalized HSC line and investigating the impact of phospholipid-based anti-fibrotic treatments on EV composition and release. The results of this study could pave new avenues towards non-invasive diagnostic tools for liver fibrosis that could help physicians in designing timely and personalized anti-fibrotic therapies at any stage of the diseases.

In another project, ribonucleic acid (RNA)-based therapeutics, including small interfering RNA (siRNA), microRNA (miRNA), messenger RNA (mRNA), and CRISPR (clustered regularly interspaced short palindromic repeats) and CRISPR-associated protein 9 (Cas9) components, play an important role. These RNA-based therapeutics have unprecedented therapeutic potential [106] and hold the promise of treating any disease with a genetic component. However, inefficient delivery into diseased cells hinders their clinical progress. Consequently, there is an urgent need for novel, original approaches to overcome the delivery challenges. Recently, an endogenous RNA transport system has emerged, based on the release and uptake of EVs or exosomes [107,108,109]. However, reproducible methods for efficient loading of EVs with therapeutic RNA are lacking. Here, the group of Raymond Schiffelers (University Medical Center Utrecht, Utrecht, the Netherlands) comes into play. The project of Schiffelers aims to improve loading of EVs with therapeutic RNA through fusion with liposomes by developing a novel method to fuse EVs with RNA-loaded liposomes and demonstrating the efficacy of these liposome–EV hybrids for therapeutic RNA delivery. Both should finally result in a prototype of a new generation RNA delivery system based on liposome–EV hybrids.

A third project is also related the drug loading of exosomes but partially in relation to stem cell therapy. Stem cell therapy is investigated in the management of difficult-to-treat or as of yet incurable diseases, such as cardiac infarction, graft-versus-host disease, and Alzheimer’s disease. With few exceptions, the application of living cells for therapeutic purposes has not yet fully found its way into standard clinical practice, in part due to safety issues and an unclear mode of action. Instead, stem cell-derived exosomes have emerged as key mediators of stem cell biological effects and additionally allow for the elimination of the safety concerns associated with living cells. Furthermore, the inherent therapeutic efficacy of exosomes [110] can be improved by encapsulation of appropriate drugs within their structure, thereby rendering them excellent candidates to substitute cell-based therapies in the future. Although exosomes have been intensely exploited as drug delivery vehicles derived from natural sources, there is only limited access to efficient and mild drug loading procedures, especially for hydrophilic drugs [111,112,113,114]. Here, the aim of the project of Jean-Christophe Leroux (ETH Zurich, Zurich, Switzerland) is to engineer exosomes with enhanced anti-inflammatory activity. To achieve this goal, a robust method for the reproducible purification of functionally preserved stem cell exosomes will be optimized and validated. Subsequently, a loading method will be established allowing the efficient encapsulation of the anti-inflammatory hydrophilic model-drug pentoxifylline into these exosomes, while preserving their integrity. Therefore, a novel loading strategy based on the fusion of exosomes with drug-loaded liposomes will be investigated. Different fusogenic lipid classes and liposomal formulations thereof will be tested to optimize the fusion and, consequently, the drug loading efficiency. With this explorative and fundamental project, Leroux aims at establishing a platform technology for the drug loading of exosomes.

#### 3.1.4. Other Liposomal Approaches for Parenteral Administration

Besides the above-mentioned projects using stimuli-responsive liposomes, targeted liposomes, or exosomes, there are four more projects dealing with phospholipid liposomes.

In the project of Federico Bordi and Simona Sennato (La Sapienza University of Rome and CNR-ISC, Rome, Italy), a new liposome-based multi-drug delivery system is developed, loading simultaneously two anti-*Mycobacterium tuberculosis*-drugs, formed by different liposomes glued together in “multi-compartment” clusters [115]. The innovation connected both with the modular structure of the carrier and with the possibility to transport and deliver different drugs simultaneously towards the same target, encapsulating them in the different liposomes forming the aggregates. It is recognized that inhalation of drug-loaded liposomes offers potential value in anti-tuberculosis (TB)-therapy. Multicompartment liposomal clusters, with sizes larger than a single vesicle, have been demonstrated to possess an intrinsic selectivity towards macrophages [116], thus representing an emerging platform for the intracellular delivery of anti-TB drugs to the primary site of infection. The opportunity of combining an increased efficacy of intracellular delivery with the ability of carrying different APIs, simultaneously and with a controlled stoichiometry, to the same target could represent a significant breakthrough.

The production of liposomes with high encapsulation efficiency for high molecular weight APIs, which are sensitive towards heat and aggressive conditions of pH, organic solvents, or surfactants, is an unmet need of increasing interest. Here, the group of Hermann Nirschl (Karlsruhe Institute for Technology, Karlsruhe, Germany) developed a novel process using centrifugation of water-in-oil (w/o) emulsions, based on the use of phospholipids as emulsifiers, avoiding the use of aggressive treatments and allowing asymmetric membrane functionalities. While the previous project addressed the characterization of the applied substances to produce liposomes [55,117,118,119,120,121], the follow-up study focuses on the optimization of liposome production process [122,123]. The proof-of-concept of the flotation of aqueous droplets from a w/o nano-emulsion to an aqueous phase by centrifugation shows advantages when compared to known liposome production methods; the use of solvents is redundant, and the encapsulation efficiency of hydrophilic model substances is higher than anything described in the literature so far. As the development is driven by engineering aspects, process parameters such as the influence of temperature and centrifugal force are examined. The key elements of this study bear strong potential for large scale industrial applications.

Another project is focused on non-alcoholic steatohepatitis (NASH). Non-alcoholic fatty liver disease (NAFLD), closely associated with obesity and metabolic syndrome affecting 20–35% population worldwide, is the leading indication for liver transplantation. NASH is the most aggressive form of NAFLD that progresses to end-stage liver failure and liver cancer, and there is no curative therapy available [124]. Lipidomic analyses in NASH patients have revealed the accumulation of “toxic” lipids such as saturated fatty acids, free cholesterol, and more complex lipids, and deficiency of “healthy” lipids, which are PUFAs and PUFA-derived specialized pro-resolving mediators (SPMs). Furthermore, high-fat diet induced a disproportional increase in full-length (inflammatory) over truncated (pro-resolving) oxidized phospholipids (OxPLs) [125,126,127]. Since SPMs and truncated OxPLs are decreased during NASH, it is hypothesized that replenishment of these bioactive lipids would be a highly promising approach for the treatment of NASH. However, these specialized bioactive lipids are poorly water-soluble and usually administered in ethanol in (pre)clinical studies. Hence, the aim of the project from Ruchi Bansal (University of Twente, Enschede, the Netherlands) is to incorporate these pro-resolving lipids in liposomes to improve their solubility and bioavailability. Overall, this project will address the pro-resolving activity of these “bioactive” liposomes in NASH and may open new therapeutic opportunities for the treatment of other chronic diseases [128].

Lastly, the project of Enrico Mastrobattista (Utrecht University, Utrecht, the Netherlands) aims to lay the foundation for the use of liposomes to stimulate bone regeneration (osteogenesis) by means of mild immune activation. This is nourished by the knowledge that a short-lived inflammation is pivotal for normal bone healing after damage, and by recent findings that local induction of a mild inflammation can lead to de novo bone formation. Factors such as liposome charge and type of phospholipid will be addressed regarding their intrinsic immuno-stimulatory and osteogenic effects. Furthermore, the functionalization of liposomes with synthetic ligands for pattern-recognition-receptors (PRRs), which could enhance their inflammatory activity, will be investigated. PRR modulation has led to clinical efficacy in anticancer drugs or vaccines. However, the usage for musculoskeletal disease is still in its infancy, and it is unknown which PRR ligands have the greatest osteogenic potential. In addition, it is unclear how (functionalized) liposomes must be applied at the biomaterial–tissue interface. At the practical level, this project strives to demonstrate the feasibility of liposome-stimulated osteogenesis by fabricating a liposome-based implant coating.

#### 3.1.5. Further Lipid-Based Formulations for Parenteral Administration

In addition to the large number of liposome-based projects, there are also several studies that use lipid-based formulations for the intended parenteral application, but focusing on other “lipid structures”, such as lipodisks or extrudates, or by pursuing completely different approaches. An overview about these parenteral projects is given in Table 4. For projects approved prior 2017, please refer to our homepage (www.phospholipid-research-center.com).

In this category, the first project is related to cancer therapy. Nanocarriers based on cationic lipids have been largely investigated to deliver DNA or RNA in different forms of cancer. Self-assembling nanoparticles (SANPs) based on cationic lipids have been recently developed for the delivery of bisphosphonates in different form of tumors [129,130], among them glioblastoma [131]. In this respect, the group of Giuseppe De Rosa (University Federico II, Naples, Italy) proposes to test SANPs to deliver miRNA in a novel therapeutic approach against glioblastoma. As a first result, selected SANP compositions based of the cationic lipid 1,2-dioleoyl-3-trimethylammonium-propane chloride (DOTAP) in combination with Cer-PEG (a PEGylated sphingosine-based lipid) and 1,2-distearoyl-*sn*-glycero-3-phosphoethanolamine-*N*-(amino(polyethylene glycol)_2000_) (DSPE-PEG_2000_), respectively, showed ideal physical–chemical characteristics in terms of size and polydispersity index, high miRNA encapsulation efficiency, good stability following incubation in serum, and no hemolytic activity [132]. Moreover, these formulations allowed enhanced miRNA uptake to be achieved, especially in a U87MG (glioblastoma) cell line, compared to the control and to the other tested formulations. The formulation containing the Cer-PEG showed the highest miRNA intracellular uptake. Further studies could clarify the mechanism by which formulations containing Cer-PEG could provide highest transfection into the cells as well as higher miRNA delivery in some organs, such as the brain.

PC-bound ω3 fatty acids (ω3-PC) suppress homeostatic pathways with fundamental roles in tumorigenesis, metastasis, and tumor resistance. By attenuating pro-survival Akt activation, ω3-PC delays cell cycle progression and enhances apoptosis. ω3-PC also increases membrane rigidity, impairs cell migration, interferes with cytoskeleton dynamics, and serves as substrate for the biosynthesis of tumor-suppressive lipid mediators. Supported by the PRC, the potential of ω3-PC in overcoming Akt-dependent tumor resistance has been explored by the group of Andres Koeberle (University Innsbruck, Innsbruck, Austria) in an earlier project, and it has been found that the cytotoxic activities of distinct bioinspired agents are potentiated by the combination with ω3-PC [133,134,135]. The actual project is intending to find a renewable and cheap source of ω3-PC, develop a bioactive formulation, and initiate pre-clinical studies on murine leukemia. By screening 23 macro- and microalgae, they recently identified a cost-efficient source of ω3-PC and will now establish a standardized procedure to obtain ω3-PC-rich fractions. The project will explore the (pre) clinical potential of ω3-polyunsaturated phospholipids in supportive anti-leukemic therapy and provide fundamental insights into their physiologically relevant molecular mechanisms.

Controlled parenteral drug delivery presents many advantages compared to conventional parenteral formulations. Along with biodegradable polymers, phospholipids have been widely applied in this field, resulting in several commercially available formulations primarily based on liposomes [8]. Solid implants present several advantages compared to colloidal phospholipid formulations, namely the ease of manufacture, drug molecule stability during the manufacturing process (e.g., protein or drugs prone to hydrolysis), as well as stability during storage. Additionally, the use of lipid-based solid implants avoids such drawbacks as microenvironmental acidification, and, potentially, interactions with incorporated proteins, which may lead to protein aggregation and incomplete release, compared to poly (lactide-*co*-glycolide) (PLGA). In this respect, Marina Kolbina and Roland Bodmeier (Freie Universität Berlin, Berlin, Germany) are exploring twin-screw extruded phospholipid-based implants for controlled drug delivery [136]. After they showed that implants can be prepared by hot melt extrusion of HSPC [137], this follow-up study is intended to define the critical process and formulation parameters and to demonstrate the applicability of phospholipid-based implants for parenteral controlled delivery both for small molecule drugs and model proteins—to present natural, biodegradable excipients as an alternative to synthetic biodegradable polymers.

In a similar context, the project of Karsten Mäder and Annette Meister (Martin Luther University Halle-Wittenberg, Halle/Saale, Germany) is about to develop and characterize anti-inflammatory phospholipid depot formulations including PS- and PG-enriched extrudates and nanofibers, respectively, for the local therapy in brain, ear, and eye. The project is based on the encouraging results [138] obtained with PC extrudates for controlled release [139] and the biological activity of PS and PG-containing phospholipid nanodispersions. PS [140]- as well as PG [141,142]-containing phospholipid depot systems have high potential for the local treatment of inflammation. To gain detailed insights into the drug delivery mechanism, Mäder and Meister will use electron spin resonance (ESR) spectroscopy and multispectral optical imaging to characterize micro-mobility, micro-polarity, and pH value. The released material will be characterized with respect to the amount, size, and structures. Both the controlled release and the anti-inflammatory properties are attractive aspects with high potential for clinical applications in terms of local therapies against inflammation.

Another project is related to contrast agents. Ultrasound contrast agents are comprised of phospholipid-coated gas microbubbles, 1–10 μm in size, that vibrate (i.e., expand and compress) in response to ultrasound. However, the vibration of current microbubbles is unpredictable and, thus, a huge problem for the new theranostic applications of microbubbles, which include ultrasound molecular imaging [143] and drug delivery [144,145]. Here, the group of Klazina Kooiman (Erasmus MC University Medical Center Rotterdam, Rotterdam, the Netherlands) aims to produce an innovative phospholipid-coated microbubble that responds on demand, controlled by ultrasound. To achieve this goal, different phospholipids must be tested to produce the microbubbles, and these microbubbles must be characterized with state-of-the-art techniques such as unique ultra-high-speed optical imaging [146] and 4Pi confocal microscopy to asses lipid miscibility and phase [147]. The new theranostic microbubbles will be evaluated in vitro and in vivo for their therapeutic drug delivery and diagnostic properties.

The last project in this subgroup is related to phospholipid nanodisks, also termed lipodisks. Katarina Edwards (Uppsala University, Uppsala, Sweden) is exploring the potential of this novel type of lipid-based nanocarrier for the formulation and delivery of (cancer) therapeutic agents. These disk-shaped nanocarriers are stable, biocompatible, and very versatile structures [148]. Due to their unique properties, the lipodisks are of interest as carriers for several different classes of anticancer agents. Previous studies showed that a formulation in lipodisks can be used as a viable means to increase tumor accumulation and reduce off-target toxicity of both the anticancer peptide melittin and chemotherapeutic drugs, such as doxorubicin and paclitaxel [149]. Recent results from the Edwards lab showed that by decorating the lipodisks with ligands that recognize selected cell membrane receptors, it is possible to design systems capable of specific tumor cell targeting and true intracellular delivery of both conventional and peptide-based anticancer drugs [150,151,152]. In this project by Edwards, the possibilities to use lipodisks for the co-delivery of chemotherapeutic drugs and membranolytic anticancer peptides will be investigated. The hypothesis is that the membranolytic peptides, apart from being toxic to the cancer cells by themselves, will help sensitize drug resistant cells to the co-administered chemotherapeutic drug. The ambition is that the knowledge gained in the project will contribute to the development of lipodisks as a versatile platform for efficient, safe, and selective delivery of anticancer agents—in addition to the liposomes used in the majority.

### 3.2. Oral Administration

When phospholipids, for example in the form of liposomes, are administered orally, one must be aware of degradation processes due the conditions found in the GI tract, i.e., low pH values and the presence of lipases and bile salts. The digestion of (phospho)lipids begins in the oral cavity through exposure to lingual lipases, which are secreted in the buccal cavity by the Ebner’s glands located on the tongue. Digestion continues in the stomach through the effects of both lingual and gastric enzymes. The stomach is also the major site for the emulsification of dietary fat and fat-soluble vitamins, with peristalsis as a major contributing factor. Crude emulsions of lipids enter the duodenum as fine lipid droplets and then mix with bile and pancreatic juice to undergo marked changes in chemical and physical structure. Emulsification continues in the duodenum along with hydrolysis and micellization in preparation for the absorption across the intestinal wall [153]. For further information on the physiological fate of phospholipids after oral ingestion, we refer to the literature [10].

Due to the phospholipids’ amphiphilic nature, they can be technically used in oral dosage forms as emulsifier, wetting agent, solubilizer (mixed micelles), and liposome former. In addition, they can be used as matrix material for solid dispersions when solubilization and fast release of the API are required, whereas saturated phospholipids may be perfectly suitable for slow release matrix tablet formulations. The main types of these formulations can be considered for optimization of the oral bioavailability of poorly water-soluble compounds [42,43].

In the following, we discuss PRC-funded projects covering the oral use of such lipid-based formulations (Table 5). For research projects approved prior 2017, please refer to our homepage (www.phospholipid-research-center.com).

Despite the success of liposomes administered parenterally, the delivery of liposomes via the oral route is impeded by various barriers, namely the chemical instability of phospholipids in the GI tract, the loss of integrity of the liposomes due to mechanical instability of the vesicles itself, and the poor permeability of conventional liposomes due to their relatively large size and the presence of various epithelial barriers in the intestine. A very promising approach to improve the chemical and mechanical stability of liposomes in the GI tract is the partly replacement of the classical monopolar phospholipids by bipolar amphiphiles, so-called bolalipids or bolaamphiphiles [154,155]. These bolalipids (natural bolalipids are also termed tetraether lipids (TELs)) could be inserted in a stretched manner in a conventional phospholipid bilayer and can act as a rivet to stabilize the bilayer and, hence, the liposome. Several groups have already shown that this approach works [100,156,157,158,159].

Bolalipids are molecules that consists of a long hydrophobic alkyl chain and two hydrophilic headgroups attached to each end. This special class of lipid molecules can be found in the membranes of certain species of Archaea, for example *Thermoacidophiles*, where they contribute to the outstanding stability of the Archaea against harsh living conditions, such as low pH values, high salt concentrations, and/or high temperatures. However, the use of the natural occurring archaeal lipids is challenging due to the high costs of the cultivation of the Archaea, and isolation of membrane lipids often results in a mixture of TELs varying in their hydrophobic parts. On the other hand, the total synthesis of these archaeal lipids is also time-consuming, expensive, and in most cases only a small amount of product can be obtained. Here, the group of Simon Drescher (Martin Luther University Halle-Wittenberg, Halle/Saale, Germany) is trying to find an easy-to-synthesize bolalipid that can be used for the stabilization of orally administered liposomes. During this project, two novel classes of artificial bolalipids, the glycerol diether bolalipids [160,161] and alkyl-substituted single-chain bolalipids [162,163], were synthesized and characterized. Drescher and his group could show that these artificial bolalipids are miscible with unsaturated phospholipids [164,165], and that these mixed vesicles (bolasomes) show promising results regarding the stability of those bolasomes in artificial digestive media [166]. However, these bolalipid-doped liposomes require further investigation before they can be used as a drug delivery system.

The project of Alexander Treusch (University of Southern Denmark, Odense, Denmark) is tackling the stability issue of orally administered liposomes from a different perspective. Instead of synthesizing artificial bolalipids or extracting them from cultured species, he is trying to exploit alternative environmental sources where bolalipid-producing Archaea are abundant. While TELs are present in a range of microorganisms of the domain Archaea, only TELs from two hyperthermophilic species have been tested for oral drug delivery by liposomes so far [159]. The main reasons are the demanding growth requirements of most TEL-producing Archaea, which make the biotechnological production of TELs uneconomical. Yet, unexplored sources of TELs are environmental Archaea, mainly *Thaumarchaeota*, which are abundant in, for example, wastewater treatment plants and the ocean. They are phylogenetically closely related to hyperthermophilic species and produce unique TELs that have evolved to stabilize the membranes at the ambient temperatures at which these organisms live, which is an interesting feature for the use in liposomes. Hence, the aim of this project is to test if environmental sources for TEL-producing Archaea can be exploited in an economical way.

Besides stabilized liposomes, one can also use oral mixed micellar formulations for drug delivery purposes. In the past, particularly in very young children, liquid dosage forms were the formulations of choice in oral (pediatric) drug administration. Although they have an excellent safety profile, little attention has been paid to the evaluation of phospholipids in pediatric formulation development. Besides ensuring patient adherence and allowing for the administration of flexible doses, pediatric formulation concepts should ensure that the drug is sufficiently bioavailable, and the pediatric dosage form is made of excipients that are proven to be non-toxic in this vulnerable patient group. These requirements are a challenge when an enabling formulation is required to guarantee a robust solubilization in the GI environment of the target patient group. The combination of natural phospholipids and bile salts in designing mixed micelle formulations represents an interesting formulation approach for developing oral pediatric dosage forms for poorly soluble drugs [167,168,169]. In the project of Sandra Klein (University Greifswald, Greifswald, Germany), natural phospholipids will specifically be screened for their applicability in oral pediatric mixed micelle formulations. The focus of the project is to establish a new phospholipid-based platform by designing a safe and effective oral pediatric dosage form, which will close an important gap in oral pediatric drug delivery and promote the use of phospholipids in pediatric formulation development.

Another project is focused on the development of phospholipid-based depot antimalarial tablets. Malaria affects millions of people yearly in malaria-endemic regions of the world, and cases of treatment failure have been recorded with artemisinin combination therapies (ACTs), for example, artemether and lumefantrine, the first-line drug combination for the treatment of *Plasmodium falciparum* malaria. Hence, design and development of more effective and affordable drug delivery systems are necessary, as drug resistance can also be combated through the engineering of novel drug delivery systems. The project of Anthony Attama (University of Nigeria, Nsukka, Nigeria) is focused on the development of a novel delivery system of an antimalarial extract clinically proven to be effective against *P. falciparum* malaria, together with long-standing use in traditional settings, and an alternative delivery system of artemether/lumefantrine combination (ACT) [170,171]. The oral phospholipid-based tablets, which will contain upwards of 50% of phospholipid, will be compared with commercial ACT. Overall, these novel formulations will increase the utilization of phospholipids as very important pharmaceutical multifunctional excipient.

Besides the use of tablets and orally administered liposomes, one can also think of amorphous solid dispersions to deliver drugs. Co-amorphous drug lecithin systems are envisaged as both amorphous solid dispersions and lipid-based drug delivery systems. As such, it is hypothesized that they form an excellent basis for both poorly water-soluble lipophilic compounds (“grease ball” molecules) and poorly water-soluble compounds with high melting points (“brick dust” molecules), and thus may be broadly applicable. In this context, the project of Thomas Rades (University of Copenhagen, Copenhagen, Denmark) is envisaged to investigate the potential use of lecithin [10] as a co-former to develop co-amorphous solid dispersions for a range of poorly water-soluble drugs [172,173,174,175,176]. Additionally, the use of co-amorphous drug lecithin systems as a new source of drug-forms to be added to lipid-based drug delivery systems (self nano-emulsifying drug delivery systems (SNEDDS)) will be investigated. This is hypothesized to be especially beneficial for “brick dust” molecules that are largely not suitable for delivery in lipid-based drug delivery systems due to poor oil solubility.

Phospholipids may also play an important role with respect to peptide/protein delivery. The oral delivery of peptides is desired in many therapeutic regimes but is challenged by the physical–chemical properties of the peptides, resulting in low absorption. Low gastric pH and proteases distributed in the entire GI tract can degrade orally administrated peptides [177,178,179]. Tight junctions connecting the enterocytes in the small intestine impede the uptake of peptides, necessitating the use of permeation enhancers in the formulation. One possible way of increasing peptide absorption after oral dosing is by lipophilization of the peptides and subsequent incorporation of the complexes into self-emulsifying drug delivery systems (SEDDS) as the final delivery system [180,181]. The SEDDS can be designed and functionalized as to have the desired properties, which are inclusion of permeation enhancers, protection of the peptide from proteolysis, delay of lipid digestion, and mucoadhesion. In the corresponding project by Anette Müllertz (University of Copenhagen, Copenhagen, Denmark), lipophilic peptide complexes using phospholipids and mono-acyl phospholipids, respectively, will be developed, and these complexes will be incorporated into SEDDS. Selected SEDDS will be tested in animal models (rats), both for assessing the mechanism behind peptide absorption and for evaluating the pharmaco-kinetics after oral dosing—to finally increase the understanding of how to enable oral peptide delivery.

The last project in this “oral” context is also related to amorphous solid dispersions. The aim of the project by Paulina Skupin-Mrugalska (Poznan University, Poznan, Poland) is the usage of phospholipids as components of mixed phospholipid and polymer-based amorphous solid dispersions for oral delivery of poorly soluble drugs. Selected formulations will be screened by using derivatives of cellulose or polyvinylpyrrolidone and (hydrogenated) phospholipids. Amorphous solid dispersions of drug substance (e.g., Fenofibrate) in a polymer-phospholipid matrix will initially be prepared by simple film-casting/batch-melting and later by hot-melt extrusion. Fenofibrate is commonly used as a model drug for amorphous solid dispersion studies and has been reported as a suitable candidate for hot-melt extrusion manufacturing [182,183]. The innovative aspect of this project embraces carriers for oral application, based on mixed matrices composed of polymers and (hydrogenated) phospholipids, in which the drug is present in an amorphous state. These carriers presumably may increase the bioavailability of the incorporated drug and have not been so far screened and characterized in detail. Therefore, it is assumed that the outcome of this work will increase the interest in the application of hydrogenated phospholipids as an excipient for oral formulations. 

### 3.3. Topical Administration

Phospholipid-based formulations have gained major interest in the field of topical drug delivery due to their physicochemical properties. As amphiphilic constituents of cellular membranes, phospholipids exhibit both high biocompatibility and great emulsifying power. Thus, plentiful phospholipid-based formulations and drug carriers such as liposomes, SLNs, micelles, and organogels have been developed and investigated by different research groups [184,185,186,187,188].

There are four projects covering the topical use of lipid-based formulations (Table 6), which are discussed below. For projects approved prior 2017, please refer to our homepage (www.phospholipid-research-center.com).

Phospholipids, especially of plant origin, are excellent excipients for dermal administration. They can be used to promote drug–skin interactions, to enhance the skin barrier, to act as a moisturizer, and to increase the time of residence of a drug substance at the skin surface. Phospholipids are further applied topically, for example in the lung, to the eye, to the vagina, etc., enabling the administration of poorly water-soluble drugs and extending the drug release, respectively. Furthermore, phospholipid liposomes are suitable for the encapsulation of hydrophilic as well as lipophilic drug substances, and these liposomes can be used in different dosage forms such as gels and liquid sprays. There are slightly different effects of phospholipids in or on the skin; whereas formulations with unsaturated phospholipids tend to penetrate deeper into the skin compared to formulations without phospholipids—thereby helping to deliver the drug to the target site—hydrogenated phospholipids, in turn, increase retention time of drugs on the surface of the skin and help to keep the barrier function intact.

A topical vaginal therapy presents the non-invasive and direct delivery of drugs to the site of action at lower doses while escaping adverse side effects caused by systemic drug administration. To achieve efficient, safe, and targeted vaginal therapy of sexually-transmitted bacterial diseases and recurrent bacterial vaginosis, the group of Željka Vanić (University Zagreb, Zagreb, Croatia) aims to develop an efficient, safe, and stable liposome-based delivery system containing azithromycin destined for improved topical vaginal therapy of sexually-transmitted diseases and bacterial vaginosis. Encapsulation into liposomes is expected to increase azithromycin solubility and allow fusion with bacterial cells, resulting in an improved localized effect even at lower doses in comparison with the free drug.

The human skin serves as a barrier between the body and the environment. Therefore, it is prone to microbial, thermal, mechanical, and chemical threats that can cause acute or chronic wounds. Triterpenes from the outer bark of birch are known for various pharmacological effects, including enhanced wound healing [189,190]. Polymeric nanofibers have been utilized to develop drug delivery systems to treat various diseases, for example medicated wound dressing [191,192]. In this respect, the project of Rolf Daniels (University Tübingen, Tübingen, Germany) aims to use birch bark dry extract to develop a bioactive nanofiber wound dressing including phospholipids as dispersants. The o/w dispersion will be blended with commercially available biodegradable and biocompatible polymers to form nanofibers intended for wound therapy using electrospinning technology. The main aim of this study is to develop a bioactive wound dressing by incorporating a sub-micron dispersion of phospholipid-stabilized birch bark dry extract into polymeric sub-micron fibers through electrospinning for wound therapy [193,194].

To increase the knowledge in the field of cytotoxicity of topical formulations and of phospholipids, the project of Claudia Valenta (University Vienna, Vienna, Austria) evaluates different phospholipid formulations (for topical use) on the cell viability of viable human keratinocytes and fibroblasts, as determined by cell culture toxicity tests. Moreover, the effect of the formulations on skin penetration and permeation of incorporated model drugs is determined [195,196]—to find an “optimal” phospholipid composition that fits nearly all requirements. The Valenta group also aspires to develop wound healing formulations based on lecithins and natural APIs, such as Betulin or Norway spruce balm. These two ingredients have been used for centuries to treat acutely and chronically infected wounds, and their incorporation into modern formulations would be a major step forward [197,198].

As mentioned above, natural plant-based phospholipids are interesting excipients for the formulation of macro- and micro-emulsions for dermatological as well as cosmetic uses. Many natural phospholipids are available for topical formulations. Although such phospholipids mixtures are well described in terms of headgroup and fatty acid composition, their physicochemical properties are not described in detail to enable a more rational selection for the effective design of topical formulations. In this context, the project of Reinhard Neubert and Gerald Brezesinski (Institute of Applied Dermatopharmacy at the Martin Luther University Halle-Wittenberg, Halle/Saale, Germany) characterizes plant-based hydrogenated (saturated) phospholipids for dermato-pharmaceutical and cosmetic use [199,200]. Therefore, phospholipids from different plant sources as well as monoacyl-PC, i.e., LPC, are physicochemically characterized in selected model systems, that are 2D monolayers, 3D dispersions, liposomes, and emulsions, using hydrophilic–lipophilic deviation (HLD) and the critical packaging parameter, solubility studies, as well as thermodynamic (differential scanning calorimetry (DSC) and isothermal titration calorimetry (ITC)), structural (small angle X-ray scattering (SAXS) and grazing incidence X-ray diffraction (GIXD)), and spectroscopic methods (total reflection X-ray fluorescence (TRXF), Fourier-transform infrared (FTIR), and Raman) for this purpose.

### 3.4. Basic Research

Finally, there are three phospholipid-based projects, which do not deal with a special form of application/administration but rather with basic research on phospholipids, although it must certainly be said at this point that the boundaries here are “fluid”. Table 7 provides an overview of these projects. For projects approved prior 2017, please refer to our homepage (www.phospholipid-research-center.com).

The first project in this category by Thomas Gutsmann and Christian Nehls (Research Center Borstel, Germany) focuses on the production of liposomes to mimic bacterial membranes using a novel microfluidic platform [201,202,203]. This technique is adapted for bacterial lipid compositions of different heterogeneity. In addition to symmetric liposomes, several approaches will be developed to produce asymmetric as well as double-membrane liposomes to mimic bacterial envelopes. One major advantage of this approach is the controlled generation of liposomes of homogenous diameters and lipid distributions. The double-membrane concept allows systems mimicking envelopes of Gram-negative bacteria to be reconstituted. The use of such systems to characterize the performance of drug candidates could help pharmaceutical companies to identify emerging resistance patterns at the very beginning of drug design and thus accelerate the development process. In addition to mimicry for drug testing, another application perspective is the functionalization of microfluidic-derived liposomes for new drug administration strategies. The use of envelope vesicles enables remarkable Trojan-horse approaches by the combination of inner and outer membrane. While the outer membrane can be customized for the targeting of bacteria, for example in biofilms, the inner membrane can be optimized for encapsulation and release of antimicrobial drugs.

The next project is really a theoretical one. Despite the generally recognized relevance of applying theoretical concepts to the design of lipid-based drug carrier systems, there is little ongoing research to develop new and further refine existing concepts. The group of Sylvio May (North Dakota State University, Fargo, USA) therefore proposes to develop a variety of theoretical models for the interaction between drug molecules and liposomes. Within this project, the influence of membrane-intercalating drug molecules on the stability of liposomes [204,205], the conformation of vesicles containing PEG-lipids studied using theory and computer simulations, the impact of drug molecules on the dynamics of vesicle formation, and the kinetics of drug release from liposomes in spatially inhomogeneous systems will be evaluated. May aims to extract and to understand the general physical mechanisms [206] that relate to the formation and stability of drug-hosting liposomes. Hence, the result of this research will be the identification of mechanistic insights and the characterization of design principles for drug-containing liposomes—re-emphasizing the importance of theoretical research in the field of phospholipids.

The last project in this context is about the fate of mixed micellar formulations after i.v. administration. Mixed micellar drug delivery systems (MM-DDS) are used to solubilize poorly soluble drugs. They are applicable for oral and parenteral administration, well tolerated, stable upon storage, and easy to produce. MM-DDS have been marketed for decades, but despite all the advantages, the number of products has remained quite limited. A reflection paper issued by the EMA [207] names several issues that impede more widespread application. The sensitivity of MM-DDS to transformations after administration is named as a key problem. The classic MM-DDS consists of a bile salt and phospholipid in an equimolar mixture. Diluting bile salt/phospholipid mixed micelles (MM) causes a release of bile salts into solution and, below a critical bile salt content in the MM, the conversion of the increasingly lipid-rich MM to liposomes. After i.v. injection, the MM-DDS get diluted and exposed to plasma proteins and blood cells. Depending on MM composition and conditions, micelles can persist, transform to liposomes, or vanish all together. Particularly the latter poses a risk of precipitation if the API cannot dissolve or bind to plasma proteins quickly enough. MM consisting of surfactants with a very low critical micellar concentration (CMC) can persist dilution but may still be affected by plasma proteins or release the API despite their persistence. In this context, the project by Heiko Heerklotz and Leonie Naßwetter (University Freiburg, Freiburg, Germany) aims to establish a better mechanistic and quantitative physicochemical understanding of MM-DDS before and after administration. This should permit insights into the parameters governing the fate of MM-DDS in vivo. It should benefit the field of lipid-based drug delivery systems by aiding a more rational and faster formulation development.

### 3.5. Upcoming Projects

In addition to the projects described above, there are some new projects that have been approved quite recently. The project by Jai Prakash (University Twente, Enschede, the Netherlands) about immunostimulating liposomes targeting the M2 macrophage to eradicate cancer follows on seamlessly from the work he has completed (see Table 3 and explanations below the table). The project by Lisa Rahnfeld (University Bern, Bern, Switzerland) is about the development of an injectable phospholipid-based depot technology for sustained drug release [208,209]. This project is a follow-up study to the finished project by Paola Luciani (ibid.). Lastly, an oral project by Meike van der Zande (Wageningen University and Research, Wageningen, the Netherlands) and Josbert Metselaar (Helmholtz Institute for Biomedical Engineering, Aachen, Germany) is investigating the behavior of liposomes as delivery systems for poorly water-soluble compounds during digestion and absorption processes.

## 4. Concluding Remarks

Despite the relatively simple chemical structure of phospholipids, this review shows how versatile the applications of phospholipids are and how little we know in some areas—which in turn is the reason for the existence and mission of the Phospholipid Research Center Heidelberg. The PRC has set itself the goal of exploring and harnessing the full potential of phospholipids. Phospholipids are already included in numerous approved drug products, but their potential is far from exhausted. Phospholipids are extremely well tolerated—they are biocompatible, biodegradable, and non-toxic—and their capabilities go far beyond those of conventional emulsifiers and solubilizers, respectively. Moreover, phospholipids have multifunctional technological properties as surfactant excipient, being, for example, emulsifier, solubilizer, liposome former, and wetting agent, and they can be applied in any route of administration. In the latter regard, they are superior compared to synthetic, non-biodegradable excipients, which are not always suitable for every route of administration and which are, per se, non-physiological.

With this review, we tried to give an overview of the current and PRC-supported research on phospholipids. As one will quickly realize, phospholipids are not the same as liposomes, and the applications of phospholipids are much more diverse. Especially in the field of oral dosage forms, the liposomal structure may not be a prerequisite for solubilizing lipophilic actives, but the phospholipids in the liposomal dosage form will be, after dissolving of the liposomes in the bile and enzymatic degradation by phospholipases, integrated into mixed micelles with bile salts and in this way enhance the solubility of the lipophilic APIs. Nevertheless, there may also be a future for intact liposomes, using bolalipids, which are not susceptible to enzymatic degradation in the gut. Such liposomes may be useful for enhancing the oral absorption of encapsulated APIs. Another important function of phospholipids is their use as emulsifiers in emulsions, especially o/w emulsions, for any route of administration. They compete in that respect for oral and topical administration with alternative synthetic detergents/emulsifiers or polymers. After parenteral administration, they also compete with solubilizing or emulsifying synthetic detergents, but phospholipids do not possess the properties to cause allergic reactions up to anaphylactic shocks, typical for these synthetic detergents, such as the polysorbates. Therefore, research on parenteral administration with phospholipids is in focus, also when taking the number of projects supported by the PRC into consideration.

Due to the advent of lipid-based gene therapy, as demonstrated by the product Onpattro, and the vigorous research ongoing in the area of lipid nanoparticles as adjuvants in COVID-19 vaccine candidates, it is to be expected that the area of phospholipids for parenteral use will be extended with many more interesting lipids, which are designed to optimize drug delivery or vaccination. Phospho-lipids may still play a role as essential, non-toxic, helper lipids as backbone for the lipid carriers. Therefore, the Phospholipid Research Center will continue its research in this innovative area, hoping that the many interesting and stimulating papers resulting from this research will represent meaningful support of drug delivery.

To convert the many exciting research findings into development projects with industrial applications, the PRC will also support feasibility study projects in the future that explore the suitability of certain research findings for further development into products, based on the use of phospholipids.

## Figures and Tables

**Table 1:** Phospholipid composition of vegetable de-oiled lecithins (derived from product specifications [16]) and egg phospholipids of different PC contents (after extraction and chromatography [17]), respectively. Data taken from [9].

Phospholipid	Lecithin and Phospholipids (% *w/w*)					
	Soybean	Sunflower Seed	Rapeseed	Egg (64–79% PC)	Egg (80–85% PC)	Egg (≥98% PC)
PC	20–22	20–26	23–31	72	81	99
PE	16–22	4–10	9–15	17	8.5	0.0
PI	13–16	15–19	15–18	-	-	-
PA	5–10	2–5	5–10	-	-	-
SM	-	-	-	2.0	2.0	0.4
LPC	<3	<3	<3	2.0	2.0	0.0
LPE	-	-	-	1.0	0.3	0.0

**Table 2:** Fatty acid composition of vegetable de-oiled lecithins (derived from product specifications [16]) and egg phospholipids of different PC contents (after extraction and chromatography [17]), respectively. Data taken from [9].

Fatty Acid	Lecithin and Phospholipids (% *w/w*)					
	Soybean	Sunflower Seed	Rapeseed	Egg (64–79% PC)	Egg (80–85% PC)	Egg (≥98% PC)
C14:0	0.1	0.1	0.1	0.2	0.1	0.2
C16:0	21	16	10	31	31	34
C18:0	4.7	5.3	0.8	15	14	12
C18:1	9.9	21	49	24	28	28
C18:2	57	54	31	16	15	16
C18:3	5.0	0.2	4.4	-	-	-
C20:0	0.1	0.3	0.1	-	-	-
C20:4	-	-	-	5.6	4.8	3.6
C22:0	0.4	1.5	0.1	-	-	-
C22:4	-	-	-	0.3	0.3	0.2
C22:5	-	-	-	0.2	0.2	0.1
C22:6	-	-	-	2.2	1.8	1.8

**Table 3:** Overview of PRC-funded research projects (2017–now) covering the parenteral use of liposomal formulations.

Principal Investigator	Host	Start/ Status	Title of Project
*Stimuli-Responsive Liposomes*			
Robbert Jan Kok	University Utrecht, the Netherlands	2017, finished	Encapsulation of plant-derived toxins in stimuli-sensitive liposomes
Tatu Lajunen	University of Helsinki, Finland	2019, ongoing	Light activated liposomes for cancer therapy
Mans Broekgaarden	University Grenoble, France	2020, ongoing	Radiation-responsive liposomes for controlled release and tumor permeation of radiotherapy dose enhancers
Heijan Xiong	University of Texas at Dallas, USA	2020, ongoing	Highly photosensitive phospholipid nanovesicles for near infrared light-triggered local anesthesia
*Targeted Liposomes*			
Luisa Corvo	University Lisbon, Portugal	2017, finished	Targeted liposomal antioxidant and anti- inflammatory therapy for liver ischemic reperfusion injury
Jai Prakash	University Twente, the Netherlands	2018, finished	Modulating tumor-associated macrophages using cell-specific targeted liposomes
Avi Schroeder	Israel Institute of Technology	2018, ongoing	Phospholipids as metastases-targeting molecules using barcoding as a new research tool in liposome discovery
Michele Bernasconi	Bern University Hospital, Switzerland	2020, ongoing	Targeted liposomal drug delivery to pediatric sarcomas: beyond the EPR effect
Joke den Haan	Amsterdam UMC, the Netherlands	2020, ongoing	Virus-like liposomes targeting CD169+ dendritic cells as a novel carrier for cancer immunotherapy
Ulrike Müller	University Heidelberg, Germany	2020, ongoing	Liposome mediated delivery of biologicals to the brain as a novel therapeutic strategy for Alzheimer’s disease
*Exosomes*			
Paola Luciani, Gregor Fuhrmann	University Bern, Switzerland and HIPS, Germany	2017, ongoing	Lipid-based therapeutics for liver fibrosis and their impact on extracellular vesicles
Raymond Schiffelers	University Medical Center Utrecht, the Netherlands	2018, ongoing	Liposome–extracellular vesicle hybrids for therapeutic RNA delivery
Jean-Christophe Leroux	ETH Zurich, Switzerland	2018, ongoing	Research on the drug loading of exosomes
*Other Liposomal Approaches*			
Federico Bordi, Simona Sennato	La Sapienza University and CNR-ISC Rome, Italy	2017, ongoing	Antitubercular drug-loaded multi-liposomes vectors
Hermann Nirschl	KIT, Germany	2019, ongoing	Encapsulation of active pharmaceutical ingredients into liposomes via centrifugation of water-in-oil nano-emulsions
Ruchi Bansal	University Twente, the Netherlands	2019, ongoing	Bioactive liposomes for the treatment of non-alcoholic steatohepatitis (NASH)
Enrico Mastrobattista	University Utrecht, the Netherlands	2020, ongoing	Liposome-based coatings for immune stimulation and bone regeneration

**Table 4:** Overview of PRC-funded research projects (2017–now) covering the parenteral use of lipid-based formulations but excluding the use of liposomes.

Principal Investigator	Host	Start/ Status	Title of Project
Giuseppe De Rosa	University Federico II, Naples, Italy	2017, ongoing	Lipid nanovectors to use non-coding RNA oligonucleotides in glioblastoma in combination with standard therapy
Andreas Koeberle	University Innsbruck, Austria	2019, ongoing	Potential of algal phosphatidylcholines containing Ω3 fatty acids in the supportive therapy of leukemia and lymphoma
Roland Bodmeier, Marina Kolbina	Freie Universität Berlin, Germany	2019, ongoing	Twin-screw extruded phospholipid implants for controlled parenteral delivery
Karsten Mäder, Annette Meister	University Halle, Germany	2020, ongoing	PS and PG enriched extrudates and nanofibers for local anti-inflammatory therapies
Klazina Kooiman	Erasmus MC University Medical Center Rotterdam, the Netherlands	2017, ongoing	Theranostic phospholipid-coated ultrasound contrast agents: response on demand
Katarina Edwards	Uppsala University, Sweden	2019, ongoing	Lipodisks for dual delivery of chemo- therapeutic drugs and anticancer peptides

**Table 5:** Overview of PRC-funded research projects (2017–now) covering the oral use of lipid-based formulations.

Principal Investigator	Host	Start/ Status	Title of Project
Simon Drescher	University Halle, Germany	2017, finished	Liposomal oral drug delivery: the use of bipolar amphiphiles to stabilize liposomes
Alexander Treusch	University of Southern Denmark	2019, ongoing	Improving nano-particulate carriers for oral drug delivery using archaeal tetraether lipids from novel sources
Sandra Klein	University Greifswald, Germany	2019, ongoing	Oral mixed micelle formulations—a novel phospholipid-based platform for safe and effective pediatric drug delivery
Anthony Attama	University of Nigeria	2018, ongoing	Development of phospholipid-based depot antimalaria tablets of *Azadirachta indica* leaf extract and artemether/lumefantrine for oral delivery
Thomas Rades	University of Copenhagen, Denmark	2019, ongoing	Co-amorphous drug–lecithin systems— bridging the gap between amorphous solid dispersions and lipid-based drug delivery
Anette Müllertz	University of Copenhagen, Denmark	2019, ongoing	Enabling oral delivery of peptides by designing phospholipid complexes for self-emulsifying drug delivery systems
Paulina Skupin-Mrugalska	Poznan University of Medical Sciences, Poland	2020, ongoing	Phospholipids as excipients in amorphous solid dispersions: an attempt to establish hot-melt-extrusion for oral formulations of poorly soluble drugs

**Table 6:** Overview of PRC-funded research projects (2017–now) covering the topical use of lipid-based formulations.

Principal Investigator	Host	Start/ Status	Title of Project
Željka Vanić	University Zagreb, Croatia	2017, finished	Synergy-based delivery system for combating sexually-transmitted bacterial infections: liposomal azithromycin-in-chitosan hydrogel
Rolf Daniels	University Tübingen, Germany	2017, finished	Electrospun bioactive wound dressing containing phospholipid stabilized nanodispersions of a birch bark dry extract
Claudia Valenta	University Vienna, Austria	2018, ongoing	Development and analysis of different phospholipid formulations for dermal application and their effect on human dermal cell viability
Reinhard Neubert, Gerald Brezesinski	Institute of Applied Dermatopharmacy, Halle/Saale, Germany	2019, ongoing	Characterization of plant-based hydrogenated phospholipids for cosmetic and dermal application

**Table 7:** Overview of PRC-funded projects (2017–now) covering basic phospholipid research.

Principal Investigator	Host	Start/ Status	Title of Project
Thomas Gutsmann, Christian Nehls	Research Center Borstel, Germany	2018, ongoing	Bottom-up designed synthetic bacteria—a tool to develop new antibiotic strategies
Sylvio May	NDSU, USA	2018, ongoing	Theoretical model to describe formation and stability of liposome–drug complexes
Heiko Heerklotz, Leonie Naßwetter	University Freiburg, Germany	2020, ongoing	Establishing a fundamental understanding of the fate of mixed micellar formulations after intravenous administration

## Abbreviations

ACT	artemisinin combination therapy
ADI	acceptable daily intake
ALA	alpha-linolenic acid;
API	active pharmaceutical ingredients
ARA	arachidonic acid
ATP	adenosine triphosphate
BBB	blood–brain barrier
Cas9	CRISPR-associated protein 9
CD44	cluster of differentiation 44;
CMC	critical micellar concentration
CPP	cell penetrating peptides
CRISPR	clustered regularly interspaced short palindromic repeats
DCC	dicyclohexylcarbodiimide
DHA	docosahexaenoic acid
DMAP	4-(dimethylamino)pyridine
DOTAP	1,2-dioleoyl-3-trimethylammonium-propane chloride
DPA	docosapentaenoic acid
DPPC	1,2-dipalmitoyl-*sn*-glycero-3-phosphocholine
DSC	differential scanning calorimetry
DSPE-PEG_2000_	1,2-distearoyl-*sn*-glycero-3-phosphoethanol-amine-*N*-amino(polyethylene glycol)_2000_
ELISA	enzyme-linked immunosorbent assay
EPR	enhanced permeability and retention
ESR	electron spin resonance
EV	extracellular vesicles
FDA	US Food and Drug Administration
FTIR	Fourier-transform infrared
GI	gastrointestinal
GIXD	grazing incidence X-ray diffraction
GPC	glycerophosphocholine
GRAS	generally recognized as safe
HA	hyaluronic acid
HLD	hydrophilic–lipophilic deviation
HSC	hepatic stellate cells
HSPC	hydrogenated soybean phosphatidylcholine
i.m.	intramuscular
i.v.	intravenous
ITC	isothermal titration calorimetry
LLOD	lower limit of detection
LPE	lyso-phosphatidylethanolamine
miRNA	micro ribonucleic acid
ML-I	mistletoe-lectin-I
MM	mixed micelles
MM-DDS	mixed micellar drug delivery systems
MNBA	2-methyl-6-nitrobenzoic anhydride
MPS	mononuclear phagocytic system
mRNA	messenger ribonucleic acid
MTP-PE	muramyl tripeptide
NAFLD	non-alcoholic fatty liver disease
NASH	non-alcoholic steatohepatitis
NIR	near-infrared
NSAID	non-steroidal anti-inflammatory drugs
o/w	oil-in-water
OXIL	oxidation-responsive liposomes
OxPL	oxidized phospholipids
PA	phosphatic acid
PC	phosphatidylcholine
PE	phosphatidylethanolamine
PEG	polyethylene glycol
PG	phosphatidylglycerol
PI	phosphatidylinositol
PLA1	phospholipase A1 (alternatively A2, B, C, D)
PLGA	poly(lactide-*co*-glycolide)
POPC	1-palmitoyl-2-oleoyl-*sn*-glycero-3-phosphocholine
PRC	Phospholipid Research Center Heidelberg
PRR	pattern-recognition-receptor
PS	phosphatidylserine
PUFA	polyunsaturated fatty acids
RNA	ribonucleic acid
s.c.	subcutaneous
SANP	self-assembling nanoparticle
SAXS	small angle X-ray scattering
SEDDS	self-emulsifying drug delivery systems
siRNA	small interfering ribonucleic acid
SLN	solid-lipid nanoparticles
SM	sphingomyelin
SNEDDS	self nano-emulsifying drug delivery systems
SPM	specialized pro-resolving mediators
TAM	tumor-associated macrophages
TB	tuberculosis
TEL	tetraether lipid
TRXF	total reflection X-ray fluorescence
TSL	temperature-sensitive liposomes
USL	ultrasound-sensitive liposomes
USP	United States Pharmacopeia
w/o	water-in-oil
WHO	World Health Organization
